# The Association Between Depressive Symptoms and the Weekly Duration of Physical Activity Subset by Intensity and Domain: Population-Based, Cross-Sectional Analysis of the National Health and Nutrition Examination Survey From 2007 to 2018

**DOI:** 10.2196/48396

**Published:** 2024-07-05

**Authors:** Josheil K Boparai, Sarah Dunnett, Michelle Wu, Vanessa K Tassone, Sophie F Duffy, Valentina Zuluaga Cuartas, Ziming Chen, Hyejung Jung, Catherine M Sabiston, Wendy Lou, Venkat Bhat

**Affiliations:** 1 Interventional Psychiatry Program St. Michael's Hospital Toronto, ON Canada; 2 Department of Biostatistics Dalla Lana School of Public Health University of Toronto Toronto, ON Canada; 3 Department of Kinesiology Faculty of Kinesiology and Physical Education University of Toronto Toronto, ON Canada; 4 Mental Health and Addictions Services St. Michael's Hospital Toronto, ON Canada; 5 Department of Psychiatry University of Toronto Toronto, ON Canada; 6 Institute of Medical Science Temerty Faculty of Medicine University of Toronto Toronto, ON Canada

**Keywords:** depressive disorder, exercise, physical activity intensity, recreational physical activity, work-related physical activity, National Health and Nutrition Examination Survey, NHANES, nutrition surveys, recreational activity, physical activity, depression

## Abstract

**Background:**

Prior literature suggests a dose-response relationship between physical activity (PA) and depressive symptoms. The intensity and domain of PA are suggested to be critical to its protective effect against depression; however, existing literature has shown mixed results.

**Objective:**

The purpose of this population-based study is to examine the associations between depressive symptoms and weekly duration of (1) total PA and (2) PA subset by intensity, domain, or both.

**Methods:**

A cross-sectional analysis of National Health and Nutrition Examination Survey data from 2007 to 2018 was conducted using multivariable logistic and linear regression models and survey weights. Participants (N=29,730) were 20 years and older and completed the Physical Activity Questionnaire and Depression Screener. The primary outcome was the presence of depressive symptoms, and the secondary outcomes were cognitive-affective and somatic symptoms of depression.

**Results:**

Participants (N=29,730) had a weighted mean age of 47.62 (SD 16.99) years, and 15,133 (51.34%) were female. On average, participants without depressive symptoms engaged in 10.87 hours of total PA per week, whereas participants with depressive symptoms engaged in 8.82 hours (*P*<.001). No significant associations were seen between the weekly duration of total PA and depressive symptom odds, somatic, or cognitive-affective symptoms (all *P*>.05). Participants with an increased weekly duration of recreational PA had decreases in depressive symptom odds (adjusted odds ratio [aOR] 0.965, 95% CI 0.944-0.986) and in somatic (adjusted coefficient [aβ]=–0.016, 95% CI –0.022 to –0.009) and cognitive-affective (aβ=–0.015, 95% CI –0.023 to –0.007) symptoms. When recreational PA was subset by intensity, participants with an increased weekly duration of vigorous-intensity recreational PA had decreases in depressive symptom odds (aOR 0.926, 95% CI 0.883-0.972) and in somatic (aβ=–0.021, 95% CI –0.032 to –0.010) and cognitive-affective (aβ=–0.022, 95% CI –0.035 to –0.009) symptoms. However, significant associations were not seen for the weekly duration of work-related, moderate- or vigorous-intensity PAs (all *P*>.05).

**Conclusions:**

Findings suggest that recreational, not work-related PA is associated with reduced symptoms of depression. Future studies should explore the impact of the different types and contexts of PA on depressive symptomatology.

## Introduction

Depression is the leading cause of disability, affecting 280 million individuals globally, with limited treatment accessibility due to stigmatization and financial barriers [1]. Physical activity (PA) can have a positive impact on mood and mental health [2] among individuals with a clinical diagnosis of depression, as well as nonclinical community subsamples [3], providing the potential for more accessible and cost-effective therapeutic modalities that improve well-being [4,5]. Further, PA can be protective against depression. A study on a community sample from Alameda County in the United States showed that individuals with low baseline levels of PA had a significantly higher risk of depression at follow-up compared to those with high baseline levels of PA [6].

PA intensity is determined by energy expenditure, which is expressed as multiples of the metabolic equivalent of task, and can be subdivided into 3 categories: light, moderate, or vigorous [7]. There is substantial variability in the literature regarding the optimal intensity for reducing depressive symptoms. Light-intensity PA has been reported to reduce depression as effectively as moderate-intensity PA among older adults [8]. Another study conducted with college students aged 15-24 years found an association between light-intensity PA and reduced depressive symptoms but not moderate- or vigorous-intensity PA [9]. A recent review suggested that moderate-intensity PA has a greater protective effect on depression than higher-intensity PA [10]. Despite these inconsistencies, the Centers for Disease Control and Prevention (CDC) recommends 150 minutes of moderate-intensity or 75 minutes of vigorous-intensity exercise per week or a combination of the 2 intensities to gain health benefits, including a reduced depression risk [7]. In line with these recommendations, a recent systematic review and meta-analysis found an inverse curvilinear association between PA and incident depression, wherein individuals with an activity volume of 150 or 75 minutes per week of moderate-intensity exercise reported a 25% and 18% lower risk of depression, respectively [11]. Similarly, one study using survey data of respondents to the Scottish Health Survey reported that 20 minutes per week of any type of PA (eg, low-intensity walking) is associated with a lower risk of psychological distress [12], while another study using data from the Swedish National March Cohort found that replacing 30 minutes of sedentary behavior per day with 30 minutes of light-intensity or moderate- to vigorous-intensity PA reduced the odds of depression by 13% and 19%, respectively [13]. This suggests that the benefits of PA may be noticeable well below the CDC’s recommended levels.

PA can also be classified by domain, wherein it can be recreational (leisure) or work-related (nonleisure). PA domain has a significant impact on a person’s psychosocial experience [14,15]. Recreational PA is considered more enjoyable, variable, and autonomous than work-related PA, which is often “obligatory, repetitive, or routine” [14]. Recreational PA also impacts one’s perceived level of control and can act as a distraction from negative preoccupations [16]. As a result, studies have found leisure-time PA to be associated with a lower prevalence of depressive symptoms compared to nonleisure PA [14,17], suggesting that work-related PA may act as a source of stress [18]. Additionally, reducing sedentary work while controlling for leisure-time PA [19] and engaging in a greater frequency of domestic activities (eg, housework and gardening) have both been associated with lower odds of psychological distress [12]. There are also studies that have found no association between work-related PA and depression [15,20]. Given these conflicting results, it is essential to further explore the association between PA domain and depressive symptomatology.

Depression is a heterogeneous disorder characterized by several phenotypic manifestations. One modality of subtyping is based on cognitive-affective (eg, negative mood) and somatic symptom (eg, fatigue) domains [21,22]. By subdividing depression into the 2 symptom domains, we can better understand the mechanisms by which PA is associated with depression and can ultimately inform public health guidelines.

This study explored the relationship between PA intensity and domain and depressive symptoms using the National Health and Nutrition Examination Survey (NHANES). We investigated three primary aims: how are depressive symptoms related to the weekly duration of (1) total PA; (2) PA, subset by intensity; and (3) PA, subset by domain? We hypothesized that participants with a higher weekly duration of total PA, moderate- and vigorous-intensity PA, or recreational PA will experience a decrease in depressive symptom odds. Our secondary aim was to investigate the association between depressive symptoms and PA, subset by intensity and domain. We hypothesized that participants with a higher weekly duration of moderate- and vigorous-intensity recreational PA, but not moderate- and vigorous-intensity work-related PA, will experience lower depressive symptom odds. In an exploratory aim, we investigated the relationship between depressive symptom subgroups and the weekly duration of total PA and PA subset by intensity, domain, or both.

## Methods

### Study Population

The study data were obtained from the 2007-2018 NHANES, a cross-sectional survey administered by the National Center for Health Statistics (NCHS) and the CDC [23]. NHANES assesses the health and nutritional status of US civilians (excluding institutionalized individuals) via a home interview and a health examination. Sample selection included counties or groups of neighboring counties as primary sampling units, followed by selection of segments within primary sampling units, selection of households within segments, and finally selection of individuals within households [23]. This study population consisted of respondents 20 years and older who completed the Physical Activity Questionnaire (PAQ) and the Mental Health—Depression Screener, which uses a standardized depression scale (ie, the Patient Health Questionnaire-9 [PHQ-9]).

### Ethical Considerations

The NHANES protocol was reviewed by the NCHS Research Ethics Review Board (protocols 2005-06, 2011-17, and 2018-01) with all participants providing informed consent prior to the interview and examination. The original consent provided by NHANES participants includes the use of their data for secondary analyses. As such, the secondary use of these data does not require additional consent from participants. The NHANES data are deidentified with the omission of all direct identifiers and characteristics that might lead to identification. This analysis did not receive approval from an institutional review board since the Data User Agreement provided by the NCHS specifies that the data in the data set can be used for statistical reporting and analysis [24].

### Exposure

Exposure variables in this study included the weekly duration of total PA and weekly duration of PA subset by intensity (moderate and vigorous), domain (recreational and work-related), or both. Each PA variable was analyzed on a continuous scale of total hours per week. For details on the questionnaire items used for the exposure variables, see Table S1 in Multimedia Appendix 1.

### Primary Outcome Measure

The primary outcome measure for this study was the sum of items 1 to 9 of the PHQ-9 from the Mental Health—Depression Screener. The PHQ-9 assessed the frequency of depressive symptoms over the past 2 weeks based on the *Diagnostic and Statistical Manual of Mental Disorders—Fourth Edition* major depressive disorder diagnostic criteria [25]. Responses were given on a 4-point Likert scale ranging from 0=not at all to 3=nearly every day. Participants were categorized as having depressive symptoms (score≥10) or not (score<10) [25]. This cutoff provides reliable sensitivity and specificity for the detection of major depressive disorder [25]. The PHQ-9 has also been shown to be a reliable measure of depression severity and has been validated [25]. As a sensitivity analysis, statistical analyses were conducted using continuous depressive symptom scores.

### Secondary Outcomes

Secondary outcomes were cognitive-affective (sum of responses to PHQ-9 items 1, 2, 6, 7, 8, and 9) and somatic (sum of responses to PHQ-9 items 3, 4, and 5) symptoms of depression [21,26]. These symptom subgroups were analyzed on a continuous scale.

### Covariates

To account for potential confounding bias in the relationship between PA and depressive symptoms, we adjusted for age [27], sex [28], race [29], education [30], marital status, socioeconomic status (ratio of family income-to-poverty threshold) [31], BMI [32], sleep time on weekdays or workdays [33], hours of sedentary activity [33], cigarette use [34], and general self-reported health condition. Age was continuous by 1-year increases. Sex was dichotomized. Race was categorized as Mexican American, non-Hispanic White, non-Hispanic Black, other Hispanic, and other race or multiracial. Education level was categorized as less than high school, high school or equivalent, some college or Associate of Arts degree, and college graduate or above. Marital status was categorized as married or living with partner, divorced or separated or widowed, and never married. The ratio of family income-to-poverty threshold was dichotomized: ≤1.3=low income and >1.3=mid-to-high income [35]. BMI was categorized as <18, 18 to <25, 25 to <30, and ≥30 kg/m^2^ [36]. Sleep time and sedentary activity were continuous by hourly increases. General self-reported health condition was categorized as poor, fair, and good or above. Cigarette use was dichotomized; individuals who answered “Yes” to “Smoked at least 100 cigarettes in life” and answered “Every day” or “Some days” to “Do you now smoke cigarettes?” on the Smoking-Cigarette Use Questionnaire were considered cigarette users, and those who answered “No” or “Not at all” to the respective questions were considered nonusers.

### Statistical Analysis

Statistical analyses were performed using R (version 4.2.1, R Foundation for Statistical Computing) and the package *survey* to account for the NHANES survey design. Mobile Examination Centre survey weights were used and divided by 6 to account for merging 6 survey cycles. Weighted means and SDs were estimated for continuous variables, and weighted proportions with unweighted frequencies were calculated for categorical variables. Continuous variables were compared using 2-tailed *t* tests, while categorical variables were compared using chi-square tests. The primary and secondary analyses for the outcome of depressive symptoms were performed using multivariable logistic regression models while adjusting for covariates. The exploratory analysis for the secondary outcomes, namely cognitive-affective and somatic symptoms scores, used multivariable linear regression models, also adjusting for covariates. The backward stepwise selection was used to develop the final multivariable models with null models including age, sex, race, and socioeconomic status. Statistical significance was established as *P*<.05, and *P* values were adjusted using the Holm method to account for multiple comparisons. Missing data were accounted for using an available case analysis. The sample size depended on data availability and as such, no a priori power calculations were performed.

## Results

### Descriptive Statistics

**Figure 1:**
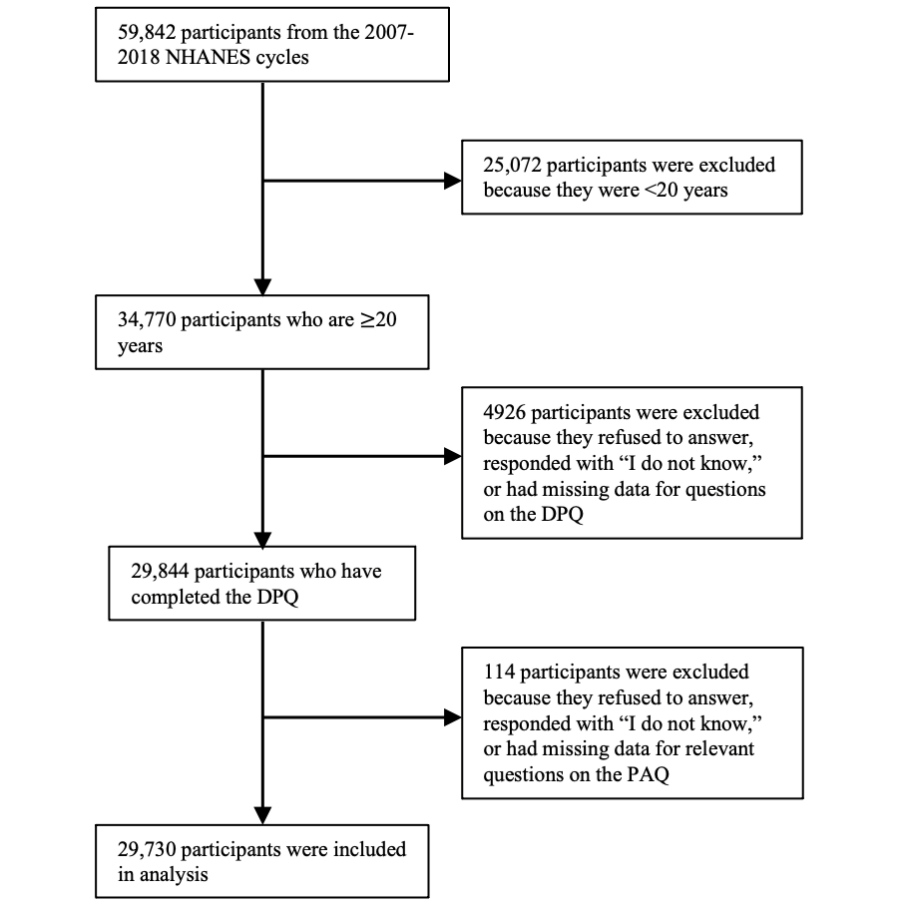
Flowchart of inclusion in the cross-sectional analyses; participants from a nationally representative sample of the United States obtained through the NHANES, 2007-2018. DPQ: Mental Health—Depression Screener; NHANES: National Health and Nutrition Examination Survey; PAQ: Physical Activity Questionnaire.

The final study sample consisted of 29,730 participants (51.34%, n=15,133, were female) aged 20-80 (mean 47.62, SD 16.99) years (refer to Figure 1 for a detailed breakdown of participant inclusion). Depressive symptom prevalence was 2739 (8.07%) participants. The descriptive statistics for each measure can be found in Table 1.

**Table 1:** Characteristics of included participants in the cross-sectional analyses from a nationally representative sample of the United States obtained through the National Health and Nutrition Examination Survey, 2007-2018^a^.

Characteristics		No depressive symptoms (<10; n=26,991)	Depressive symptoms (≥10; n=2739)	*P* value	
Age (years), mean (SD)		47.68 (17.08)	46.99 (15.94)	.15	
**Sex, n (%)**					<.001
	Female	13,385 (50.18)	1748 (64.54)		
	Male	13,606 (49.82)	991 (35.46)		
**Race, n (%)**					<.001
	Mexican American	4059 (8.52)	405 (8.03)		
	Non-Hispanic Black	5775 (10.85)	588 (13.27)		
	Non-Hispanic White	11,210 (67.39)	1157 (63.31)		
	Other Hispanic	2724 (5.59)	377 (8.01)		
	Other race or multiracial	3223 (7.66)	212 (7.38)		
**Education, n (%)**					<.001
	Less than high school	6107 (14.41)	979 (25.51)		
	High school or GED^b^	6173 (22.82)	653 (26.54)		
	Some college	8047 (31.4)	819 (33.78)		
	College and above	6643 (31.37)	285 (14.17)		
**Marital status, n (%)**					<.001
	Married or living with other	16,366 (64.59)	1208 (46.9)		
	Divorced or separated or widowed	5718 (17.34)	948 (31.22)		
	Never married	4892 (18.07)	580 (21.88)		
**PIR^c^, n (%)**					<.001
	≤1.3 (low income)	7335 (19.84)	1319 (41.89)		
	>1.3 (mid-to-high income)	17,248 (80.16)	1153 (58.11)		
**BMI (kg/m^2^), n (%)**					<.001
	<18	392 (1.42)	50 (2.12)		
	18 to <25	7259 (27.93)	590 (23.9)		
	25 to <30	8975 (33.49)	708 (26.09)		
	≥30	10,112 (37.16)	1350 (47.89)		
**Cigarette use, n (%)**					<.001
	Yes	5054 (17.85)	1014 (39.37)		
	No	21,920 (82.15)	1725 (60.63)		
**Health condition, n (%)**					<.001
	Good or above	21,403 (85.17)	1111 (49.08)		
	Fair	4989 (13.33)	1111 (36.19)		
	Poor	597 (1.50)	517 (14.73)		
**PA^d^ (hours per week), mean (SD)**					
	Total PA	10.87 (16.85)	8.82 (16.27)	<.001	
	Moderate-intensity PA	6.81 (10.98)	5.71 (10.83)	.001	
	Vigorous-intensity PA	4.06 (9.27)	3.10 (8.50)	<.001	
	Recreational PA	2.72 (4.46)	1.52 (4.02)	<.001	
	Work-related PA	8.15 (15.83)	7.30 (15.19)	.06	
	Moderate-intensity work-related PA	5.17 (10.27)	4.64 (9.98)	.07	
	Vigorous-intensity work-related PA	2.98 (8.73)	2.66 (8.09)	.17	
	Moderate-intensity recreational PA	1.63 (3.15)	1.08 (3.09)	<.001	
	Vigorous-intensity recreational PA	1.08 (2.63)	0.44 (1.92)	<.001	
PHQ-9^e^, mean (SD)		2.13 (2.39)	14.06 (3.81)	<.001	
Cognitive-affective, mean (SD)		0.78 (1.34)	7.85 (3.27)	<.001	
Somatic, mean (SD)		1.35 (1.55)	6.21 (1.94)	<.001	

^a^Unweighted frequencies are paired with weighted percentages. Mean and SDs are weighted. *P* values are based on weighted values.

^b^GED: General Educational Development.

^c^PIR: poverty-income ratio.

^d^PA: physical activity.

^e^PHQ-9: Patient Health Questionnaire-9.

### Weekly Duration of Total PA and Depressive Symptoms

After controlling for all covariates, for every 1-hour increase in the weekly duration of total PA, there was a nonsignificant decrease in the odds of depressive symptoms (Table 2) and a decrease in the somatic and cognitive-affective scores of depression (Table 3). The association with weekly duration of total PA remained nonsignificant in the sensitivity analysis (Table S2 in Multimedia Appendix 1).

**Table 2:** Weighted ORs^a^ and aORs^b^ for associations between PA^c^ and depressive symptoms for the cross-sectional analyses of participants from a nationally representative sample of the United States obtained through the National Health and Nutrition Examination Survey, 2007-2018^d^.

PA	Depressive symptoms (PHQ-9^e^≥10)			
	OR (95% CI)	*P* value	aOR (95% CI)	*P* value
Total PA	0.992 (0.987-0.996)	.002	0.996 (0.991-1.000)	.20
Moderate-intensity PA	0.990 (0.983-0.997)	.007	0.996 (0.989-1.003)	.54
Vigorous-intensity PA	0.986 (0.978-0.995)	.005	0.991 (0.983-0.999)	.14
Recreational PA	0.902 (0.877-0.928)	<.001	0.965 (0.944-0.986)	.007
Work-related PA	0.996 (0.992-1.000)	.08	0.997 (0.993-1.002)	.54

^a^OR: odds ratio.

^b^aOR: adjusted odds ratio.

^c^PA: physical activity.

^d^*P*≤.05 indicates statistical significance, and *P* values are adjusted using the Holm method. Adjusted models adjusted for the following covariates: age, sex, race, education, marital status, socioeconomic status (ratio of family income-to-poverty threshold), BMI, sleep time on weekdays or workdays, hours of sedentary activity, cigarette use, and general self-reported health condition.

^e^PHQ-9: Patient Health Questionnaire-9.

**Table 3:** Associations between PA^a^ and somatic and cognitive-affective symptoms of depression for the cross-sectional analyses of participants from a nationally representative sample of the United States obtained through the National Health and Nutrition Examination Survey, 2007-2018^b^.

PA	Somatic symptoms		Cognitive-affective symptoms	
	aβ^c^ (95% CI)	*P* value	aβ (95% CI)	*P* value
Total PA	–0.001 (–0.003 to 0.001)	≥.99	–0.002 (–0.005 to 0.000)	.12
Moderate-intensity PA	0.000 (–0.004 to 0.004)	≥.99	–0.002 (–0.005 to 0.001)	.36
Vigorous-intensity PA	–0.003 (–0.006 to 0.001)	.41	–0.004 (–0.008 to 0.000)	.12
Recreational PA	–0.016 (–0.022 to –0.009)	<.001	–0.015 (–0.023 to –0.007)	.003
Work-related PA	0.000 (–0.002 to 0.003)	≥.99	–0.001 (–0.004 to 0.001)	.36

^a^PA: physical activity.

^b^*P*≤.05 indicates statistical significance, and *P* values are adjusted using the Holm method. Adjusted models adjusted for the following covariates: age, sex, race, education, marital status, socioeconomic status (ratio of family income-to-poverty threshold), BMI, sleep time on weekdays or workdays, hours of sedentary activity, cigarette use, and general self-reported health condition.

^c^aβ: adjusted coefficient.

### Weekly Duration of PA, Subset by Intensity, and Depressive Symptoms

After adjusting for all covariates, for every 1-hour increase in the weekly duration of moderate- and vigorous-intensity PA, there was no significant association with the odds of depressive symptoms (all *P*>.05; Table 2) and the somatic and cognitive-affective scores of depression (all *P*>.05; Table 3). The association with weekly duration of moderate- and vigorous-intensity PA remained nonsignificant in the sensitivity analysis with the outcome of depressive symptoms (all *P*>.05; Table S2 in Multimedia Appendix 1).

### Weekly Duration of PA, Subset by Domain, and Depressive Symptoms

After controlling for the covariates, for every 1-hour increase in the weekly duration of recreational PA, there was a significant decrease in the odds of depressive symptoms (aOR 0.965, 95% CI 0.944-0.986; *P*=.007; Table 2) and the somatic and cognitive-affective scores of depression (aOR –0.015, 95% CI –0.023 to –0.007; *P*=.003; Table 3). The association with the weekly duration of recreational PA remained statistically significant in the sensitivity analysis (*P*<.001; Table S2 in Multimedia Appendix 1).

Covariate-adjusted models showed that there was a nonsignificant relationship between increased weekly duration of work-related PA and decreased odds of depressive symptoms (*P*=.54; Table 2). Furthermore, for every 1-hour increase in the weekly duration of work-related PA, there was no significant association with cognitive-affective and somatic scores of depression (*P*=.36; Table 3). The association with weekly duration of work-related PA remained nonsignificant in the sensitivity analysis (*P*>.99; Table S2 in Multimedia Appendix 1).

### Weekly Duration of PA, Subset by Domain and Intensity, and Depressive Symptoms

A 1-hour increase in the weekly duration of vigorous-intensity recreational PA (aOR 0.926, 95% CI 0.883-0.972; *P*=.009) was significantly associated with 7.4% lower odds of having depressive symptoms, but there was no significant association between depressive symptom odds and the weekly duration of moderate-intensity recreational PA (aOR 0.975, 95% CI 0.949-1.001; *P*=.19; Table 4). The association with the weekly duration of vigorous-intensity recreational PA (*P*=.001) and moderate-intensity recreational PA (*P*=.007) was statistically significant in the sensitivity analysis with the outcome of depressive symptoms (Table S3 in Multimedia Appendix 1). For every 1-hour increase in the weekly duration of vigorous-intensity recreational PA, the somatic (adjusted coefficient [aβ]=–0.021, 95% CI –0.032 to –0.010; *P*=.001) and cognitive-affective (aβ=–0.022, 95% CI –0.035 to –0.009; *P*=.005) scores of depression decreased significantly, but moderate-intensity recreational PA was only associated with significant decreases in somatic scores of depression (aβ=–0.016, 95% CI –0.025 to –0.007; *P*=.002). No significant associations were found for the weekly duration of vigorous-intensity work-related PA or moderate-intensity work-related PA and depressive symptoms (all *P*>.05; Table 5). The association with weekly duration of vigorous-intensity work-related PA or moderate-intensity work-related PA remained nonsignificant in the sensitivity analysis (all *P*>.05; Table S3 in Multimedia Appendix 1).

**Table 4:** Weighted ORs^a^ and aORs^b^ for associations between PA^c^ subcategories and depressive symptoms for the cross-sectional analyses of participants from a nationally representative sample of the United States obtained through the National Health and Nutrition Examination Survey, 2007-2018^d^.

PA	Depressive symptoms (PHQ-9^e^≥10)			
	OR (95% CI)	*P* value	aOR (95% CI)	*P* value
Moderate-intensity work-related PA	0.995 (0.988-1.001)	.19	0.998 (0.990-1.005)	.53
Vigorous-intensity work-related PA	0.995 (0.989-1.002)	.19	0.995 (0.987-1.002)	.35
Moderate-intensity recreational PA	0.916 (0.882-0.952)	<.001	0.975 (0.949-1.001)	.19
Vigorous-intensity recreational PA	0.829 (0.783-0.879)	<.001	0.926 (0.883-0.972)	.009

^a^OR: odds ratio.

^b^aOR: adjusted odds ratio.

^c^PA: physical activity.

^d^*P*≤.05 indicates statistical significance, and *P* values are adjusted using the Holm method. Adjusted models adjusted for the following covariates: age, sex, race, education, marital status, socioeconomic status (ratio of family income-to-poverty threshold), BMI, sleep time on weekdays or workdays, hours of sedentary activity, cigarette use, and general self-reported health condition.

^e^PHQ-9: Patient Health Questionnaire-9.

**Table 5:** Associations between PA^a^ subcategories and somatic and cognitive-affective symptoms of depression for the cross-sectional analyses of participants from a nationally representative sample of the United States obtained through the National Health and Nutrition Examination Survey, 2007-2018^b^.

PA	Somatic symptoms		Cognitive-affective symptoms	
	aβ^c^ (95% CI)	*P* value	aβ (95% CI)	*P* value
Moderate-intensity work-related PA	0.002 (–0.002 to 0.006)	.79	–0.001 (–0.004 to 0.002)	.55
Vigorous-intensity work-related PA	–0.001 (–0.005 to 0.003)	.79	–0.003 (–0.007 to 0.001)	.34
Moderate-intensity recreational PA	–0.016 (–0.025 to –0.007)	.002	–0.014 (–0.026 to –0.003)	.05
Vigorous-intensity recreational PA	–0.021 (–0.032 to –0.010)	.001	–0.022 (–0.035 to –0.009)	.005

^a^PA: physical activity.

^b^*P*≤.05 indicates statistical significance, and *P* values are adjusted using the Holm method. Adjusted models adjusted for the following covariates: age, sex, race, education, marital status, socioeconomic status (ratio of family income-to-poverty threshold), BMI, sleep time on weekdays or workdays, hours of sedentary activity, cigarette use, and general self-reported health condition.

^c^aβ: adjusted coefficient.

## Discussion

### Principal Findings

This study investigated the association of the weekly duration of PA (including subsets by intensity, domain, or both) with the overall presence of depressive symptoms and somatic and cognitive-affective depression symptomatology. The results of this study indicate that it is the PA domain, specifically recreational PA, that may be crucial to the relationship between PA and depressive symptoms. Depressive symptom odds decreased significantly with an increase in the weekly duration of recreational PA. When intensity was examined alone, neither vigorous- nor moderate-intensity PA was significantly associated with depressive symptoms. When domain and intensity were considered together, only an increase in vigorous-intensity recreational PA duration was significantly associated with a decrease in the odds of depressive symptoms. Notably, based on the demographic data collected in this study, participants with depressive symptoms were more likely to be female, have a higher BMI, have lower income, and participated in lower weekly durations of PA regardless of being subset by intensity and domain. This coincides with prior studies that have shown gender [37], BMI [32], income status [38], and PA to be associated with the likelihood of depression. As a result, to address potential confounding bias, we incorporated these variables as covariates when examining the association between the weekly duration of PA and depressive symptoms.

Our findings coincide with results from previous studies that found a significant association between decreased recreational PA and greater depressive symptoms. In addition to the neurobiological changes, such as an increased release of endorphins and neurotransmitters like dopamine and norepinephrine [39-41], the protective effect of recreational PA involves social aspects and enjoyability of recreational activities [14]. Recreational activities can be social or self-focused, both providing time structure [42-44]. Social recreational PA offers an added benefit of pleasurable social interactions with others, which can provide a distraction from negative life events and increase perceptions of social support [43,44]. Alternatively, self-focused recreational PA can be conducted individually or within a group setting and buffers against negative events through personal transformation [43]. Thus, it may be expected that recreational PA has a more comprehensive effect on depression with reduced cognitive-affective and somatic depressive symptoms. Our results demonstrated that recreational PA has similar associations with both symptom subgroups, suggesting that its impact is not limited to changes in one’s psychosocial experience. The influence on somatic symptoms may be due to recreational PA frequently occurring outside, being more enjoyable, and producing euphoric feelings that are captured as somatic [45].

In examining the statistically significant associations between the different aspects of PA and depressive symptoms, it is important to note the statistical issue that the increased power in a large sample study leads to narrower CIs for the effect measures and smaller *P* values. However, by adjusting the statistical significance using the Holm method, the chances of false positives were minimized. Acknowledging that this can still cause one to claim impractical significance for very small effects, we prioritized clinical significance over statistical significance.

While work-related PA has been associated with higher levels of depression [17], our results did not reach statistical significance. This could be attributed to sample differences, as previous research examined adults aged 50 and older [17], whereas our sample size consisted of participants aged 20-80 years. Since most work-related PAs entail heavy lifting and strenuous overuse of the neck and back without adequate rest and recovery [46], it may act as a burden for all adults, including older adults, and contribute to higher depression levels. As this study included both younger and older individuals aged 20-80 years, the impact of work-related PA on depressive symptoms may be influenced by additional factors such as job satisfaction and job type [47,48]. Additionally, in this study, work-related PA encompassed paid and unpaid work, household chores, and yard work. Given that research has found a greater frequency of domestic activities, such as housework and gardening, to be associated with lower odds of psychological distress [12], our findings should be interpreted with caution, as the 2 types of work cannot be distinguished based on the NHANES questions. Furthermore, it is important to explore an individual’s interest and competence in executing workplace PAs. Prior research suggests that within the work domain, PA may be associated with limited personal choice and is typically driven by the demands of work output and the flow of work, which can be strenuous [49]. Another study highlights the significance of occupation type, shedding light on how work-related PA can vary across different job categories [48]. For example, women in trade, labor, or transport-related jobs who engage in work-related walking experienced lower levels of psychological distress, but this association was not present when considering moderate or vigorous PAs [48]. This suggests that the nature of the job and the type of PA involved are linked to mental health outcomes. Further investigation into this will help parse out the mechanism by which work-related PA is associated with depressive symptoms.

Regular PA participation can provide a sense of mastery, improved body image, sense of achievement, and feelings of control that may distract one from negative thoughts [16]. However, this study found no significant association between the weekly duration of total PA and depressive symptom odds, which challenges previous literature that found a greater frequency of PA has a protective effect on depressive symptoms [50,51]. This contradiction may be attributable to the absence of low-intensity PA duration in the total PA calculations due to its unavailability in the NHANES data. As low-intensity PAs involve reduced energy expenditure than moderate or vigorous-intensity PA [7], they may be easier to engage in more frequently and thus show important associations with depressive symptoms. In fact, previous studies have found that light and moderate intensities of PA were both associated with lower likelihoods of depression [8,52].

Contrary to previous research [53,54], we found that hourly increases in the weekly duration of moderate- and vigorous-intensity PA did not significantly decrease depressive symptom odds. Previous research shows intensity-dependent physiological responses caused by increased frequencies of higher-intensity PA, all of which correlate with reduced depressive symptoms [55]. These physiological responses include the adaptation of the hypothalamic-pituitary-adrenal axis activity leading to increased stress resiliency [56], increased neurogenesis from elevated brain-derived neurotrophic factor expression [57], and reduced inflammation creating an anti-inflammatory environment [40,55]. The discrepancy between our study and prior literature may be attributed to the fact that our PA variables are defined using weekly duration rather than frequency. Therefore, participants with the same weekly duration of moderate-intensity or vigorous-intensity PA may be engaging in a different PA frequency (eg, 50 minutes per day for 3 days vs 30 minutes per day for 5 days). Prior research has shown that <10 minutes of exercise contributes to minimal mood improvements, whereas a longer duration (>30 minutes) can contribute to fatigue and withdrawal-related responses [58].

### Limitations

This study is not without limitations. The exposure and outcome variables were measured using self-report questionnaires, which are subject to participant biases, including recall, social desirability, and nonresponse bias. This study also used secondary data and was subject to limited predefined variables. As such, although data regarding PA domains were available, we did not have information on respondents’ activities within recreational or work-related PA. Recreational PA encompasses sports, fitness, and brisk walking, whereas work-related PA encompasses paid, unpaid work, and domestic activities. Additionally, our analysis did not consider light-intensity PA since it was not available in the NHANES questionnaire. Finally, the cross-sectional design of the study limits the conclusions to correlational rather than causal. There is a bidirectional association between PA and depression, wherein it is possible that there is a reverse causal relationship called the inhibition hypothesis [59,60], which suggests that depression symptoms such as anhedonia, low mood, and lack of energy may inhibit individuals from engaging in PA due to a lack of interest or motivation [59].

### Conclusions and Future Directions

This study demonstrates that PA domain, specifically recreational PA, might play an integral role in the protective effect of PA on depressive symptoms. Future studies should further investigate the impact of different types of recreational PA (social and self-focused) and their impact on depressive symptoms. While we found that an increased weekly duration of work-related PA was not significantly associated with lower depressive symptoms, future studies should explore the different types of occupational and household-based PA to further elucidate which activities have a detrimental effect and which have a beneficial effect. Furthermore, occupational PA can be further subdivided into paid and unpaid work to examine their effects on depressive symptoms, as these are likely to be driven by different motivations.

## Appendix

Multimedia Appendix 1 Detailed methodology and statistical analysis results.

## Abbreviations

aβ: adjusted coefficient
aOR: adjusted odds ratio
CDC: Centers for Disease Control and Prevention
NCHS: National Center for Health Statistics
NHANES: National Health and Nutrition Examination Survey
OR: odds ratio
PA: physical activity
PAQ: Physical Activity Questionnaire
PHQ-9: Patient Health Questionnaire-9

## Data Availability

The data sets analyzed during this study are available via the NHANES website [61].
