# Supplementary material for: The Association Between Depressive Symptoms and the Weekly Duration of Physical Activity Subset by Intensity and Domain: Population-Based, Cross-Sectional Analysis of the National Health and Nutrition Examination Survey From 2007 to 2018
Source: Interact J Med Res. 2024 Jul 5;13:e48396. doi: 10.2196/48396 (PMC11259767; doi:10.2196/48396)
Supplement: Multimedia Appendix 1 [file ijmr_v13i1e48396_app1.docx]

**Table S1.** Questionnaire items used for the exposure variables in the cross-sectional analyses of participants from a nationally representative sample of the United States obtained through the National Health Health and Nutrition Examination Survey (NHANES), 2007-2018.

| **Exposure Variable** | **PA Variable** | **NHANES Items** |
| --- | --- | --- |
|  |  |  |
| Domain | Work-related PA (hours/week) | (PAQ610 [number of days/week vigorous-intensity work-related PA] * PAD615 [number of minutes/day vigorous-intensity work-related PA])/60 minutes  +  (PAQ625 [number of days/week moderate-intensity work-related PA] * PAD630 [number of minutes/day moderate-intensity work-related PA])/60 minutes |
| Domain | Recreational PA (hours/week) | (PAQ655 [number of days/week vigorous-intensity recreational PA] * PAD660 [number of minutes/day vigorous-intensity recreational PA])/60  +  (PAQ670 [number of days/week moderate-intensity recreational PA] * PAD675 [number of minutes/day moderate-intensity recreational PA])/60 minutes |
| Intensity | Moderate-Intensity PA (hours/week) | (PAQ625 [number of days/week moderate-intensity work-related PA] * PAD630 [number of minutes/day moderate-intensity work-related PA])/60 minutes  +  (PAQ670 [number of days/week moderate-intensity recreational PA] * PAD675 [number of minutes/day moderate-intensity recreational PA])/60 minutes |
| Intensity | Vigorous-Intensity PA (hours/week) | (PAQ610 [number of days/week vigorous-intensity work-related PA] * PAD615 [number of minutes/day vigorous-intensity work-related PA])/60 minutes  +  (PAQ655 [number of days/week vigorous-intensity recreational PA] * PAD660 [number of minutes/day vigorous-intensity recreational PA])/60 minutes |
| Total | Total PA (hours/week) | Moderate-intensity PA (hours/week)  +  Vigorous-intensity PA (hours/week)  OR  Work-related PA (hours/week)  +  Recreational PA (hours/week) |

***Note:*** All PA variables were defined as follows by NHANES. Vigorous-intensity work-related PA was defined as paid or unpaid work, household chores, and yard work that caused large increases in breathing or heart rate, including carrying or lifting heavy loads, digging or construction work. Moderate-intensity work-related PA was defined as activities that caused small increases in breathing or heart rate, such as brisk walking or carrying light loads. Vigorous-intensity recreational PA excludes work and transport activities and includes sports, fitness and recreational activities that cause large increases in breathing or heart rate, like running or basketball. Moderate-intensity recreational PA refers to sports, fitness, or recreational activities that cause small increases in breathing or heart rate, such as brisk walking, bicycling, swimming, or volleyball.

**Table S2.** Weighted and adjusted coefficient estimates for associations between PA and depressive symptoms for the cross-sectional analyses of participants from a nationally-representative sample of the United States obtained through the National Health and Nutrition Examination Survey (NHANES), 2007-2018.

| **Depressive Symptoms** | | | | |
| --- | --- | --- | --- | --- |
| **PA** | **β (95% CI)** | ***P* Value** | **aβ (95% CI)** | ***P* Value** |
|  |  |  |  |  |
| Total PA | -0.009 (-0.013,  -0.005) | <0.001 | -0.003 (-0.007, 0.001) | 0.396 |
| Moderate-Intensity PA | -0.010 (-0.017,  -0.004) | 0.004 | -0.002 (-0.009, 0.004) | >0.999 |
| Vigorous-Intensity PA | -0.014 (-0.021,  -0.008) | <0.001 | -0.007 (-0.014,  -0.001) | 0.121 |
| Recreational PA | -0.089 (-0.104,  -0.075) | <0.001 | -0.031 (-0.044,  -0.017) | <0.001 |
| Work-Related PA | -0.003 (-0.007, 0.002) | 0.213 | -0.001 (-0.006, 0.004) | >0.999 |

*Note: P* ≤ 0.05 indicates statistical significance, p-values are adjusted using Holm’s method; aβ is adjusted coefficient estimate. Adjusted models adjusted for the following covariates; age, sex, race, education, marital status, socioeconomic status (SES) (ratio of family income-to-poverty threshold), body mass index (BMI), sleep time on weekdays or workdays, hours of sedentary activity, cigarette use and general self-reported health condition.

**Table S3.** Weighted and adjusted coefficient estimates for associations between PA subcategories and depressive symptoms for the cross-sectional analyses of participants from a nationally-representative sample of the United States obtained through the National Health and Nutrition Examination Survey (NHANES), 2007-2018.

| **Depressive Symptoms** | | | | |
| --- | --- | --- | --- | --- |
| **PA** | **β (95% CI)** | ***P* Value** | **aβ (95% CI)** | ***P* Value** |
|  |  |  |  |  |
| Moderate-Intensity Work-Related PA | -0.004 (-0.010, 0.003) | 0.505 | 0.001 (-0.006, 0.008) | 0.852 |
| Vigorous-Intensity Work-Related PA | -0.005 (-0.012, 0.003) | 0.505 | -0.004 (-0.011, 0.003) | 0.487 |
| Moderate-Intensity Recreational PA | -0.088 (-0.108,  -0.068) | <0.001 | -0.030 (-0.049,  -0.011) | 0.007 |
| Vigorous-Intensity Recreational PA | -0.132 (-0.156,  -0.109) | <0.001 | -0.043 (-0.064,  -0.022) | 0.001 |

*Note:* *P* ≤ 0.05 indicates statistical significance, p-values are adjusted using Holm’s method; aβ is adjusted coefficient estimate. Adjusted models adjusted for the following covariates; age, sex, race, education, marital status, socioeconomic status (SES) (ratio of family income-to-poverty threshold), body mass index (BMI), sleep time on weekdays or workdays, hours of sedentary activity, cigarette use and general self-reported health condition.
